# Genetic introgression from commercial European pigs to the indigenous Chinese Lijiang breed and associated changes in phenotypes

**DOI:** 10.1186/s12711-024-00893-8

**Published:** 2024-04-02

**Authors:** Ruifei Yang, Siqi Jin, Suyun Fang, Dawei Yan, Hao Zhang, Jingru Nie, Jinqiao Liu, Minjuan Lv, Bo Zhang, Xinxing Dong

**Affiliations:** 1 College of Animal Science and Technology, Yunnan Agricultural University, Kunming, China; 2 College of Animal Science and Technology, China Agricultural University, Beijing, China

## Abstract

**Background:**

Gene flow is crucial for enhancing economic traits of livestock. In China, breeders have used hybridization strategies for decades to improve livestock performance. Here, we performed whole-genome sequencing of a native Chinese Lijiang pig (LJP) breed. By integrating previously published data, we explored the genetic structure and introgression of genetic components from commercial European pigs (EP) into the LJP, and examined the impact of this introgression on phenotypic traits.

**Results:**

Our analysis revealed significant introgression of EP breeds into the LJP and other domestic pig breeds in China. Using a haplotype-based approach, we quantified introgression levels and compared EP to LJP and other Chinese domestic pigs. The results show that EP introgression is widely prevalent in Chinese domestic pigs, although there are significant differences between breeds. We propose that LJP could potentially act as a mediator for the transmission of EP haplotypes. We also examined the correlation between EP introgression and the number of thoracic vertebrae in LJP and identified *VRTN* and *STUM* as candidate genes for this trait.

**Conclusions:**

Our study provides evidence of introgressed European haplotypes in the LJP breed and describes the potential role of EP introgression on phenotypic changes of this indigenous breed.

**Supplementary Information:**

The online version contains supplementary material available at 10.1186/s12711-024-00893-8.

## Background

Genetic introgression yields a diverse range of distinct phenotypes in livestock. Unlike adaptive introgression, which occurs naturally and helps develop resistance and adaptation to environmental changes [1–3], human-mediated genetic introgression is a recent and intentional intervention related to domestication and migration of livestock [4–6]. Human-mediated genetic introgression enhances genetic diversity and improves the performance of economic traits. Over the past few decades, crossbreeding between different pig breeds facilitated by humans has greatly enhanced productivity in the pig industry, especially in terms of resource utilization.

Domestic pigs (*Sus scrofa* L.) in East Asia and Western Eurasia originated from two independent domestication events, each yielding distinct phenotypes [7, 8]. Importation of Chinese pig breeds into Europe during the Industrial Revolution led to significant introgression of Asian haplotypes into European pigs (EP) [4, 9]. These included the *EDNRB* variant associated with coat colour, the *LEMD3* gene related to ear morphology [10], the *AHR* locus related to sow fertility [4], and the *VRTN* gene related to vertebra number, carcass length, and teat count [11]. In recent decades, adoption of commercial EP breeds in China has increased gene flow between European and Chinese domestic pig populations, resulting in greater heterosis and economic value of EP compared to local breeds [12]. Previous research has documented three distinct waves of introgression from Europe to Asia over the past 200 years [13]. In addition, EP introgression may have induced phenotypic changes in indigenous Chinese pigs, such as introgression of the European *NR6A1* haplotype, which can increase the number of vertebrae, body size, and feed-conversion efficiency [14, 15] and of the *KIT* alleles from the Large White (LW) breed into the Pudong white pig, which has resulted in expression of the white coat colour phenotype [16]. Furthermore, transmission of EP-introgressed segments among Chinese pig populations may also have occurred in China in recent decades [12].

The Lijiang pig (LJP) is an ancient native breed that is primarily found in the vicinity of Lijiang City, Yunnan Province, China. It is renowned for its multi-rib characteristic and its ability to withstand cold temperatures. LJP pigs are mainly raised in free-ranging herds in remote mountainous regions and have not undergone significant genetic improvement [17]. With the advancement of artificial insemination and transportation, EP have been steadily introduced to the Lijiang region, which raises the possibility of genetic introgression and the emergence of phenotypic changes in the LJP breed. However, no systematic research has been conducted on the genomic structure of the LJP and, thus, whether EP introgression has occurred remains unclear.

In this study, we used whole-genome resequencing data from 44 LJP and previously published data to explore potential introgression from EP into LJP. We also conducted comparative analyses with other indigenous Chinese breeds and examined the effect of EP introgression on the number of thoracic vertebrae in LJP, to evaluate the potential influence of EP haplotypes on phenotypic differences in indigenous Chinese breeds.

## Methods

### Sample preparation

Blood samples were collected from the ear vein of 44 unrelated LJP. All individuals were raised and managed under consistent conditions from birth to adulthood (12 months) in a nucleus conservation field. Before slaughter, all adult pigs were humanely stunned with electricity and the number of thoracic vertebrae was recorded after dissection.

### Genome resequencing

Genomic DNA was extracted using a standard phenol–chloroform extraction method. For genome resequencing, a minimum of 0.5 μg of genomic DNA from each sample was used to construct a library with an insert size of 150 bp. Paired-end sequencing libraries were constructed and sequenced on an Illumina HiSeq X Ten Sequencer (Illumina, San Diego, CA, USA) and MGISEQ-2000 (BGI, Shenzhen, China) according to the manufacturers’ instructions.

### Sequence alignment and variant calling

We used the default parameters of the fastp software (v0.23.2) (https://github.com/Open GENE/FASTP) to filter the original data [18]. After quality control, an average of 27.35 × high-quality clean data was obtained (see Additional file 1: Table S1). Alignment was performed using the BWA software with default parameters [19]. Sscrofa11.1 was retrieved from the Ensembl database and used as the reference genome (GenBank accession number: GCA_000003025.6). We followed the optimal mutation detection and analysis process recommended on the GATK website [20], including marking duplicate reads, local realignment, and base quality recalibration. The GATK (v4.0.9) selection command ‘select variants’ was used to select single nucleotide polymorphisms (SNPs). Quality control filtration parameters were set as follows: QD < 2.0, MQ < 40.0, FS > 200.0, SOR > 3.0, mqranksum < − 12.5, and readposranksum < − 20. After the removal of SNPs on the sex chromosomes and on unknown chromosomes, 38,164,258 SNPs remained. Further quality control filters retained autosomal SNPs with a minor allele frequency > 5%, a missing rate < 95%, and those that passed the Hardy–Weinberg equilibrium test at *p* ≥ 1^–4^, which resulted in 26,358,572 SNPs for further analysis. The dataset was then combined with public data released by PigVar (VCF files) (http://202.200.112.245/pigvar/files/) [21, 22]. These public data included 47 pigs from five commercial European breeds (EP: Duroc: DUROC, Large White: LW, Landrace: LR, Pietrain: PT, and Hampshire: HAMP) and European wild boar (WBE), 11 domestic pigs from four regions in Southwest China (i.e. Southwestern Chinese domestic pigs (SWCDP): Pengzhou: PZ, Neijiang: NJ, Wujin: WJ, and Yanan: YN), 50 Tibetan pigs (TIBP) from eight regions (Daocheng: DCTIB, Litang: LTTIB, Ganzi: GZTIB, and Aaba: ABTIB, which were distributed in the Sichuan Province, China; Shangri-La: SLTIB and Diqing: DQTIB in the Yunnan Province, China; Gongbujiangda: GBTIB in the Tibet Autonomous Region, China; Gansu: GSTIB, in the Gansu Province, China) (http://iswine.iomics.pro/pig-iqgs/iqgs/index) [23], 21 domestic pigs mainly distributed in the Zhejiang or Jiangsu Province of Eastern China (i.e. Eastern Chinese domestic pigs (ECDP): Meishan: MS, Erhualian: EHL, Jinhua, JH; Jiquhai, JQH), 18 pigs from Northern Chinese domestic breeds (Northern Chinese domestic pigs (NCDP): Laiwu, LWH; Min, MZ; Hetao, HT), 20 pigs from four Southern Chinese domestic breeds (Southern Chinese domestic pigs (SCDP): Bamaxiang, BMX, Wuzhishan: WZS, Luchuan: LC, and Xiang: Xiang), 17 Asian wild boars, and two Sumatran pigs. Data were combined as follows: (1) removing SNPs with no reference SNP ID (dbSNP rsID) for public data and updating the coordinates of each SNP from Sscrofa10.2 to Sscrofa11.1; (2) removing SNPs for the two Sumatran pigs for which at least one individual had a missing genotype; (3) merging VCF files of processed public data and LJP data based on SNP rsID using the bcftools (v1.8) software; and (4) ensuring that individuals had an SNP missing rate lower than 5%. The Beagle v5.2 software was then used to impute missing SNP genotypes [24]. In total, 230 individuals with 2,409,528 variants were retained, with a marker density of one SNP per 939 bp, including 47 pigs from EP and WBE, 11 pigs from SWCDP, 50 pigs from TIBP, 21 pigs from ECDP, 18 pigs from NCDP, 20 pigs from SCDP, 17 Asian wild boars, and two Sumatran pigs (see Additional file 1: Table S1).

### Phylogenetics and population structure

Matrices of identity-by-state (IBS) and p distances between individuals were calculated using the PLINK (v1.9) (-distance square 1-ibs) and VCF2Dis software (v1.50) (https://github.com/hewm2008/VCF2Dis) [25]. These matrices were then used to construct unrooted neighbour-joining (NJ) trees using FastME (v2.1.6.1) [26]. The ‘ggtree’ package in R was used for tree visualisation [27]. We used PLINK (v1.9) to prune the SNP data based on linkage disequilibrium (LD) with the parameter ‘-indep-pairwise 50 10 0.2’. Principal component analysis (PCA) for the 33 breeds was performed on the LD-pruned data using the genome-wide complex trait analysis (GCTA) software (v1.26.0) [28], discriminant analysis of principal components (DAPC) was conducted using the R package ‘adegenet’ [29], and population admixture analysis was performed using the ADMIXTURE software (v1.3.0) [30], with K = 2 to 10; cross-validation error was obtained from each run. Admixture results were plotted using the R package ‘Pophelper’ [31].

### *D*-statistic test, *f*4 ratio, outgroup-*f*3 ratio, and topology analysis of genetic introgression between commercial European lines and indigenous Chinese pig groups

To confirm genome-wide admixture between EP and indigenous Chinese pig groups (including SWCDP; TIBP; ECDP; NCDP; and LJP; see Additional file 1: Table S1), we used *D*-statistic test, *f*4 ratio, outgroup-*f*3 ratio, and topology analysis to detect global genetic introgression signals. Based on the full set of 2,409,528 phased SNPs, we used the Dsuite software (v0.5 r52) to perform the *D*-statistic test, with a Chinese indigenous pig group as P1 [32], other closely related Chinese pig groups as P2, EP as P3, and the Sumatran wild boar as an outgroup (O). We calculated the *D*-statistic for the three P1, P2, and P3 groups and the outgroup O as ((P1, P2), P3, O), where the choice of using Sumatran wild boar as outgroup followed the procedure of Bosse et al. [4]. A positive *D*-statistic with a Z-score > 3 was used as evidence that there was significant gene flow between the P2 and P3 groups. All suitable combinations and their corresponding *D*-statistic values were plotted. The *f*4 ratios were also calculated using Dsuite and plotted along with the *D*-statistic values. We also computed the outgroup-*f*3 ratio using ADMIXTURE (v1.3.0), with a Chinese indigenous pig group as P1, EP as P2, and Sumatran wild boar as the outgroup (O) ((P1, P2), O). For topology analysis of the EP introgression, we used the Twisst software (v0.2) to calculate the topology weightings for each input tree (50 SNPs per window): (O, ((P1, P2), EP)); (O, ((P1,EP), P2)), and (O, (P1, (P2, EP))) [33], with P1 as NCDP, TIBP-E, SWCDP or LJP, and P2 as SCDP, and Sumatran wild boar as the outgroup (O).

### Local ancestry inference

Based on the aforementioned analysis, we obtained global genetic introgression signals using the *D*-statistic test, *f*4 ratio, and outgroup-*f*3 ratio statistics. Local ancestry of the genome haplotypes was further inferred using the Loter software (v1.0.1) [34], which quantifies introgressive hybridisation of genomic segments. For the Loter analysis, the reference population consisted of genetic material from the target population. The SCDP and EP groups were used as the reference population, and the LJP, NCDP, or the representative of the Tibetan pigs with introgression from EP (TIBP-E) (combining the DQTIB and DCTIB groups) were used as the target population. To verify LJP introgression into TIBP-E, the LJP and SCDP pigs were used as the reference population and TIBP-E as the target population. We counted the proportion of genome haplotypes identified as local ancestry from EP with a window size of five SNPs. If one site was identified, the entire 5-SNP haplotype was treated as local ancestry, where ‘1’ refers to the haplotype from EP and ‘0’ to the haplotype from the other reference group, SCDP. Then, we used the following ratio as the proportion of EP introgression ($P(EP)$) for a given target population (such as NCDP or LJP), which represented the EP introgressed level of each 5-SNP window bin:

$$P\left(EP\right)=\frac{N(EP)}{N\left(EP\right)+N(SCDP)},$$

where $N(EP)$ and $N(SCDP)$ represent the number of haplotypes identified to be of local ancestry from EP and SCDP, respectively.

We computed the proportions of introgression from EP in 481,898 bins and Z-transformed the proportions. A bin with a Z-score > 2 was used as evidence of significant introgression from EP. Adjacent bins that were also identified as introgressed were combined into a block. To study whether non-EP introgression segments of LJP were present in TIBP-E, we fetched window bins with an introgressed proportion exceeding 95% and removed bins that had been detected as EP introgression in TIBP-E. After completing the inference of local ancestry, we compared introgression levels between indigenous Chinese pig populations.

### Comparison of introgression from EP into NCDP, LJP, and TIBP-E breeds

We conducted an analysis to compare the NCDP, LJP, and TIBP-E breeds. First, we computed the total, N50, N40, N30, N20, and N10 block size of EP introgression segments. Subsequently, we computed the EP introgressed proportions in the NCDP, LJP, and TIBP-E groups for each 5-SNP window bin, which represented the proportion of individuals that carried a given significant introgressed segment in each population. Then, we compared the distribution of the overall EP introgressed proportions in the NCDP, LJP, and TIBP-E groups. This included the thickness (representation of the overall EP introgressed variation) in the distribution of introgressed proportions throughout the whole genome and discrepancy (representation of the specificity of the EP introgressed bins) in the introgressed regions of the LJP, NCDP, and TIBP-E groups. Then, we calculated kernel densities based on the proportion of introgression in two populations within their significantly EP introgressed regions (e.g., the proportion of introgression in the LJP compared to that in the significantly introgressed region of the TIBP-E), representing the similarities of introgressed proportions. Finally, we computed the Kullback–Leibler (K–L) divergence in the two populations using the proportion of introgression, with only one population having a significantly introgressed bin region. For example, the K–L divergence between LJP and TIBP-E was based on a significantly introgressed bin region obtained from LJP or TIBP-E, with a lower K–L divergence corresponding to greater similarity between the introgressed proportions of the two populations.

### Detection and comparative analysis of direct and indirect introgression from EP into TIBP-E pigs

Considering that there was extensive gene flow among Chinese pigs, the introgressed EP haplotype could be transmitted to each other. To explore these potential multiple transmissions of EP introgression among Chinese pigs, we classified EP introgression into direct and indirect categories. Direct introgression represented haplotypes that were directly transmitted from EP into Chinese pigs, whereas indirect introgression indicated that EP haplotypes were transmitted from a mediator into Chinese pigs. To detect indirect EP introgression and to assess the similarity of EP introgression into LJP and into TIBP-E, we used the Loter (v1.0.1) software to analyse a genomic segment identified as the local ancestry component from EP (Z-score > 2). In this analysis, LJP (or TIBP-E) and EP were used as reference populations and TIBP-E (or LJP) as the target population. Because we only detected significant bins (Z-score > 2) for introgression from LJP into TIBP-E (no significant bins were detected for the reverse), downstream analyses were based on the aforementioned Loter analysis results. To avoid incomplete lineage sorting, we Z-transformed the proportions and a bin with a Z-score > 2 indicated significant introgression from LJP. Then, by overlapping the regions of bins with introgression from EP into LJP, we identified 51 bins as possible indirect introgressions from the EP (with LJP as the potential mediator). To detect direct EP introgression, we retrieved the top 51 bins identified as introgressed segments from EP (Z-score > 2) but not from LJP (Z-score < -2), considering them as possible cases of direct EP introgression. We also based retrieval of bins from TIBP on the aforementioned Loter analysis results of EP introgression into TIBP-E and then used TIBP with no significant EP introgression (GZTIB, LTTIB, and SLTIB) and LJP as the reference population and TIBP-E as the target population. We retrieved the top 51 bins identified as the representation of genomic segments from TIBP (Z-score > 2). After retrieving bins of direct EP introgression, indirect EP introgression, and of genomic segments from TIBP in TIBP-E, we performed site-pi (genetic diversity) analysis using VCFtools (v0.1.16) based on the SNPs located within these bins [35] and used the Duncan test for multiple comparison analysis of average site-pi of SNPs located within the bins of direct EP introgression, indirect EP introgression, and of genomic segments from TIBP in TIBP-E.

Based on the identified introgression bins (n = 51) from LJP or EP into TIBP-E (possible direct or indirect introgression), first we computed haplotype frequencies and removed bins if the haplotype frequencies were lower than 5% in all pig populations. This resulted in 252 and 356 haplotypes retained for possible indirect and direct introgression, respectively, for downstream analysis. Then, we calculated the K–L divergence based on haplotype frequencies in the regions of possible direct or indirect introgression between EP and Chinese indigenous pig populations and performed t-distributed stochastic neighbour embedding (t-SNE) based on haplotype frequencies to measure the distance between pig populations. The Euclidean distance was computed using the t-SNE results between EP and SWCDP, LJP, NCDP, and TIBP-E. Using the TreeMix (v1.13) software, a maximum-likelihood tree was constructed based on SNPs in all regions of possible direct or indirect introgression [36]. To compare genetic differences in possible direct or indirect EP introgression into TIBP-E, we computed the genome-wide fixation index (Fst) at each SNP within the 51 bins that were identified between TIBP-E and EP, between LJP and EP, or between TIBP-E and LJP using VCFtools (v0.1.16) [35, 37].

### Local introgression in relation to thoracic vertebra number in LJP

To determine whether EP introgression into LJP is correlated with thoracic vertebra number, the 44 LJP individuals were divided into two groups: one with individuals that had more than 14 vertebrae (LJM, n = 27) and the other with individuals that had less than 14 vertebrae (LJL, n = 17). Then, we computed the introgressed proportion in the LJL and LJM groups based on the results of the Loter local-ancestry analysis using SCDP and EP as the reference groups and LJP as the target group in bins of five SNPs. Fisher’s exact test was used to determine whether there were significant differences between LJL and LJM and the resulting *p* value was corrected using the R package ‘fdrtool’, with the significance threshold set to FDR < 0.05 [38]. To validate the identified genetic differences for significant bins between the LJL and LJM groups, we performed a locus-specific branch-length (LSBL) analysis using SNPs within the detected bins [39] by computing the LSBL value as:

$$LSBL=\frac{({d}_{LJL-LJM}+{d}_{LJL-EP}-{d}_{LJM-EP})}{2},$$

where ${d}_{LJL-LJM}$, ${d}_{LJL-EP}$, and ${d}_{LJM-EP}$ are the pairwise Fst values between the LJL and LJM, LJL and EP, and LJM and EP groups, respectively. The haplotype block with a high genetic differentiation as well as a significant difference of EP introgression between LJL and LJM was determined based on the level of LD between the SNPs with the highest LSBL value and all flanking sites, where the boundary was verified when the LD value between the SNP with the highest LSBL value and the flanking sites was drastically decreased. The level of LD was computed using the PLINK software (v1.9) with the parameter “-ld-window-kb 2000 -ld-window 15,000 -r2 –ld-window-r2 0”. Finally, based on the defined LD block, haplotypes were retrieved from the different pig groups and haplotype frequencies were calculated. A one-tailed t-test was performed between the two LJP groups based on thoracic vertebra number, with and without dominant EP haplotypes (i.e. the haplotypes that showed the highest proportion in EP).

## Results

### Population structure and genetic relationship between LJP and other populations

**Fig. 1:**
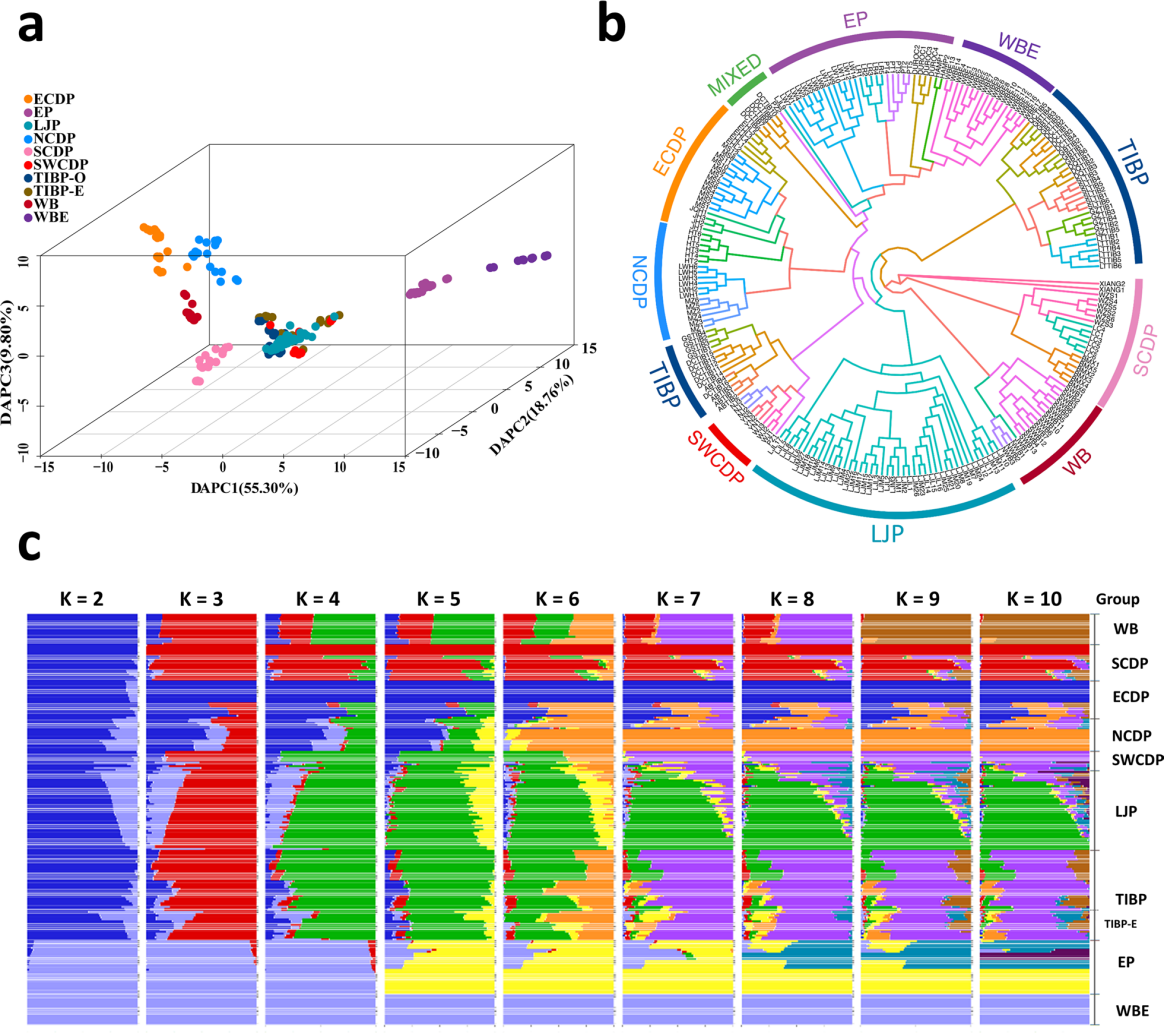
Discriminant analysis of principal components (DAPC), phylogenetic structure, and population admixture of LJP and other breeds. **a** DAPC of pig breeds using whole-genome SNPs. DAPC1, DAPC2, and DAPC3 explained 55.43%, 19.98%, and 9.00% of the genetic variance, respectively. The populations were clustered together according to their location, including Eastern Chinese domestic pigs (ECDP), Northern Chinese domestic pigs (NCDP), Southwestern Chinese domestic pigs (SWCDP), Tibetan pigs (including Tibetan pigs located in Diqing and Daocheng, i.e. TIBP-E; and other Tibetan pigs, i.e. TIBP-O), Lijiang Pig (LJP), Southern Chinese domestic pigs (SCDP), wild boar (WB), commercial European pigs (EP), and European wild boar (WBE). **b** Neighbour-joining (NJ) tree based on the IBS distance matrix of all pigs. The branch colors represent unique breed codes as described in Additional file 1: Table S1. **c** Admixture results for K = 2 to 10

In this study, we generated whole-genome sequence data from LJP which, combined with previously published data, corresponded to 230 individuals (see Additional file 1: Table S1). The pig groups were geographically classified (see Methods). Based on the SNPs generated from whole-genome sequencing data, discriminant analysis of principal components (DAPC) was performed on the 228 individuals (excluding the Sumatran outgroup), which showed that DAPC1 distinguished the EP and WBE from the Chinese indigenous breeds, while DAPC2 and DAPC3 were similar based on geographic location, including ECDP and NCDP, or SCDP and NCDP (Fig. 1a). We also conducted PCA to investigate the genetic relationships between populations, and found similar results to those of the DAPC. Specifically, LJP clustered together with SWCDP and TIBP, indicating their genetic similarity and proximity in Southwest China (see Additional file 2: Fig. S1). Furthermore, an NJ tree was constructed based on the identity-by-state (IBS) matrix and showed that LJP formed an independent branch (Fig. 1b), while several indigenous Chinese pigs were found to be closely related to EP, including NCDP (LWH, HT, and MZ), DCTIB, MS, and LJP (see Additional file 2: Fig. S2). The NJ tree and heatmap based on p-distance displayed the same topological structure (see Additional file 2: Fig. S3), further confirming the close genetic relationship between these breeds and the commercial European lines.

### Population admixture of commercial European lines and LJP

To investigate the possibility of gene flow events between the EP and LJP populations, first we conducted a population admixture analysis for the number of ancestors, i.e. K = 2 to 10 (Fig. 1c). At K = 2, the genetic composition of LJP showed a wide range of proportions of genetic component from Chinese pigs (52.5–93.0%). Similarly, certain indigenous Chinese groups, such as the Tibetan pigs from Daocheng and Diqing (DCTIB and DQTIB: 55.7–94.0%), and some SWCDP breeds (PZ: 60.3–80.6%, and WJ: 60.3–94.2%), also exhibited variable levels of purity. The NCDP showed the lowest purity compared to the other populations (NCDP (MZ: 65.9–72.2%; LWH: 73.9–81.3%; and HT: 77.0–83.5%). Several pigs with an extremely low proportion of genetic component from Chinese pigs, including PZ, DCTIB, and LJP individuals (52.5–67.3%), showed the closest relationship with EP based on the NJ tree (Fig. 1b; see Additional file 2: Fig. S3). Based on the result for K = 6, which showed the lowest cross-validation error (0.512) (see Additional file 2: Fig. S4), the EP could be distinguished from WBE in the EP group, while LJP displayed a relatively low purity with the EP genetic component (Fig. 1c). Consequently, we hypothesized that the LJP were introgressed from EP, as well as other Chinese pig groups, including NCDP, and some SWCDP and TIBP.

Although TIBP clustered together with LJP and SWCDP (Fig. 1a), it is diversely distributed in China, including the Gansu, Sichuan, Yunan, and Xizang Provinces. We observed a discordant intra-group genetic component from EP in TIBP based on the population admixture analysis. Therefore, we split the TIBP into eight groups according to their location. The *D*-statistic showed no significant introgression from the EP into GZTIB, LTTIB, and SLTIB. Hence, we used these pigs as sister groups to compare EP introgression levels for other TIBP. Accordingly, DQTIB and DCTIB exhibited the highest *D*-statistics among the TIBP groups (see Additional file 1: Table S2 and Additional file 2: Fig. S5). The admixture results also indicated that they shared more EP genetic components than the other Tibetan pigs (Fig. 1c). Consequently, we combined them to represent TIBP with EP introgression, i.e. TIBP-E. Compared to SWCDP and Tibetan pigs, the EP introgression level in LJP was comparable to that of DCTIB and higher than that of the other pig groups (see Additional file 2: Figure S5). In addition, we detected significant EP introgressed signals in NCDP (see Additional file 1: Table S2). Therefore, based on these results, we detected significant EP introgression into LJP, TIBP-E, SWCDP, and NCDP. To verify these results, we used the outgroup-*f3* statistic to compare different Chinese indigenous groups to EP and found that, compared to WB and SCDP, LJP, NCDP, TIBP, and SWCDP were more closely related to EP (see Additional file 2: Fig. S5), while LJP and NCDP showed the highest level of outgroup-*f3* statistics. Based on the topology analysis, we observed an excess number of average weighting ratios for the topology tree where EP and LJP, NCDP, SWCDP, or TIBP-E clustered together compared to the other two topologies (see Additional file 2: Fig. S6), which reflects the relatively close relationship between EP and these populations.

### Genome-wide pattern of EP introgression into LJP and other populations

Although significant introgression signals were observed in the SWCDP group, the number of individuals in each breed was not sufficient for further analysis (see Additional file 1: Table S1). As a result, the introgression level may be significantly imbalanced in this group. Therefore, the SWCDP group was not included in subsequent analyses. Using Loter analysis (see Methods), we detected 15,481, 15,273, and 14,826 EP-introgression bins at significant levels (Z-score > 2) in the NCDP, TIBP-E, and LJP, respectively (see Additional file 1: Tables S3–S5).

**Fig. 2:**
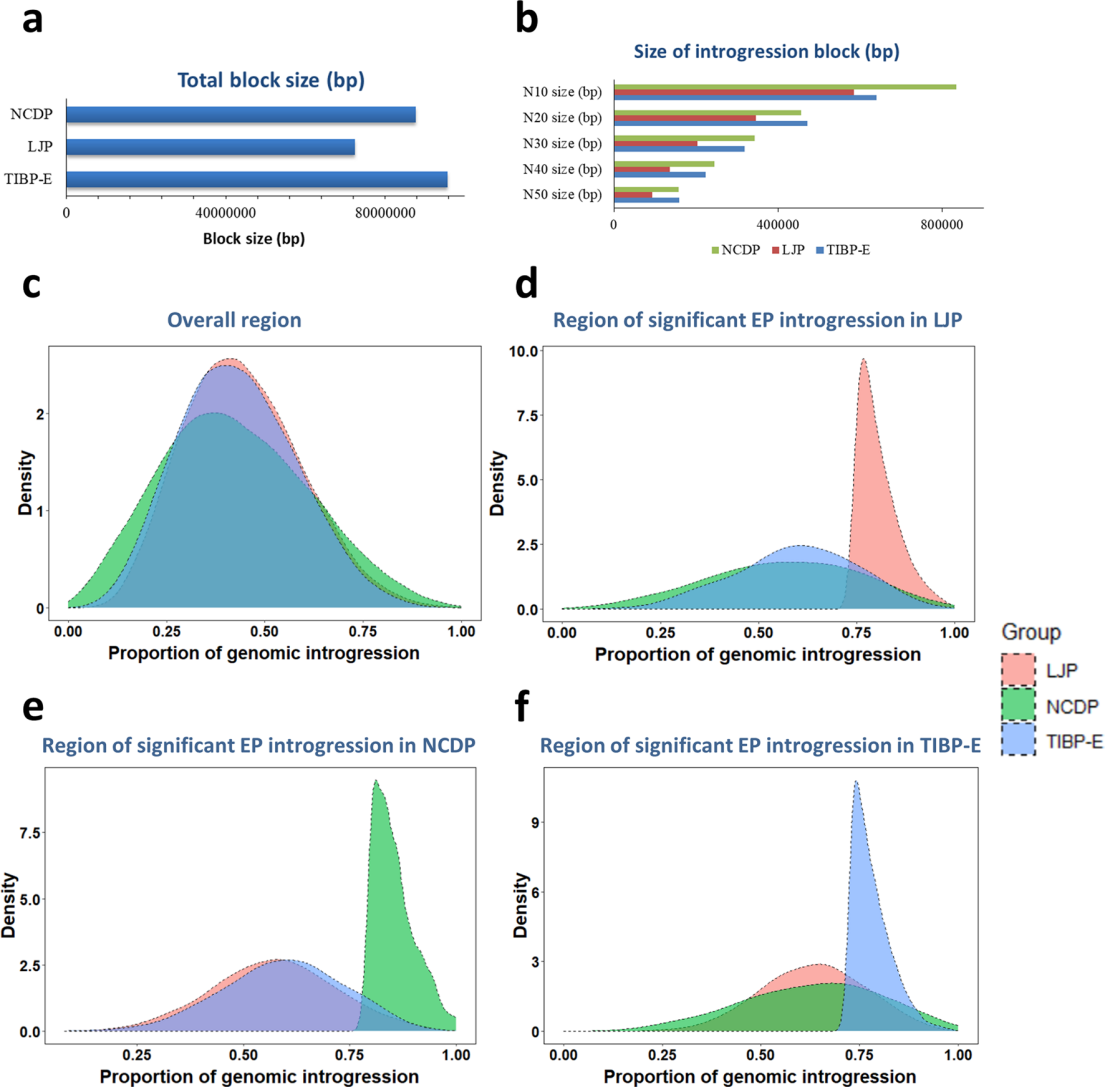
Comparisons of genome-wide introgression maps from EP for NCDP, LJP, and TIBP-E (DCTIB and DQTIB). **a** and **b** Summary of the introgression block sizes of EP for NCDP, LJP, and TIBP-E. **c** Density plot of the overall proportion of introgression from EP for LJP, NCDP, and TIBP-E with 5-SNP bins. **d** Density plot of the proportion of EP introgression into LJP, NCDP, and TIBP-E based on the region of significant EP introgression bins in LJP. **e** Density plot of the proportion of EP introgression into LJP, NCDP, and TIBP-E based on the region of significant EP introgression bins in NCDP. **f** Density plot of the proportion of EP introgression into LJP, NCDP, and TIBP-E based on the region of significant EP introgression bins in TIBP-E

We compared the size of the introgressed blocks in different populations, which showed that the EP-introgressed blocks in TIBP-E (total: 95.72 Mb; N50: 158.32 kb) and NCDP (total: 87.67 Mb; N50: 157.11 kb) were both larger than those in LJP (total: 72.46 Mb; N50: 92.88 kb) (Fig. 2a and b). To account for the impact of sample size, we down-sampled LJP (n = 18) and observed a similar small introgression block size (total: 68.56 Mb; N50: 113.25 kb) (see Additional file 2: Fig. S7). Therefore, we generated the density plot for the proportion of introgression from EP into LJP, NCDP, and TIBP-E according to genome-wide bins of five SNPs. The introgressed proportions followed a normal distribution for all groups (Fig. 2c), although the distributions for LJP and TIBP-E were narrower than those for NCDP, indicating a more uneven distribution of EP introgression across the entire genome in NCDP. Next, we generated the density plot for the EP introgressed proportions according to the significant introgression bins (Z-score > 2) in LJP, NCDP, and TIBP-E (Fig. 2d–f). This analysis showed different density distributions among the three groups within each significant introgressed regions, highlighting differences in EP introgression in LJP, NCDP, and TIBP-E.

### LJP as a potential mediator for transmitting EP haplotypes

**Fig. 3:**
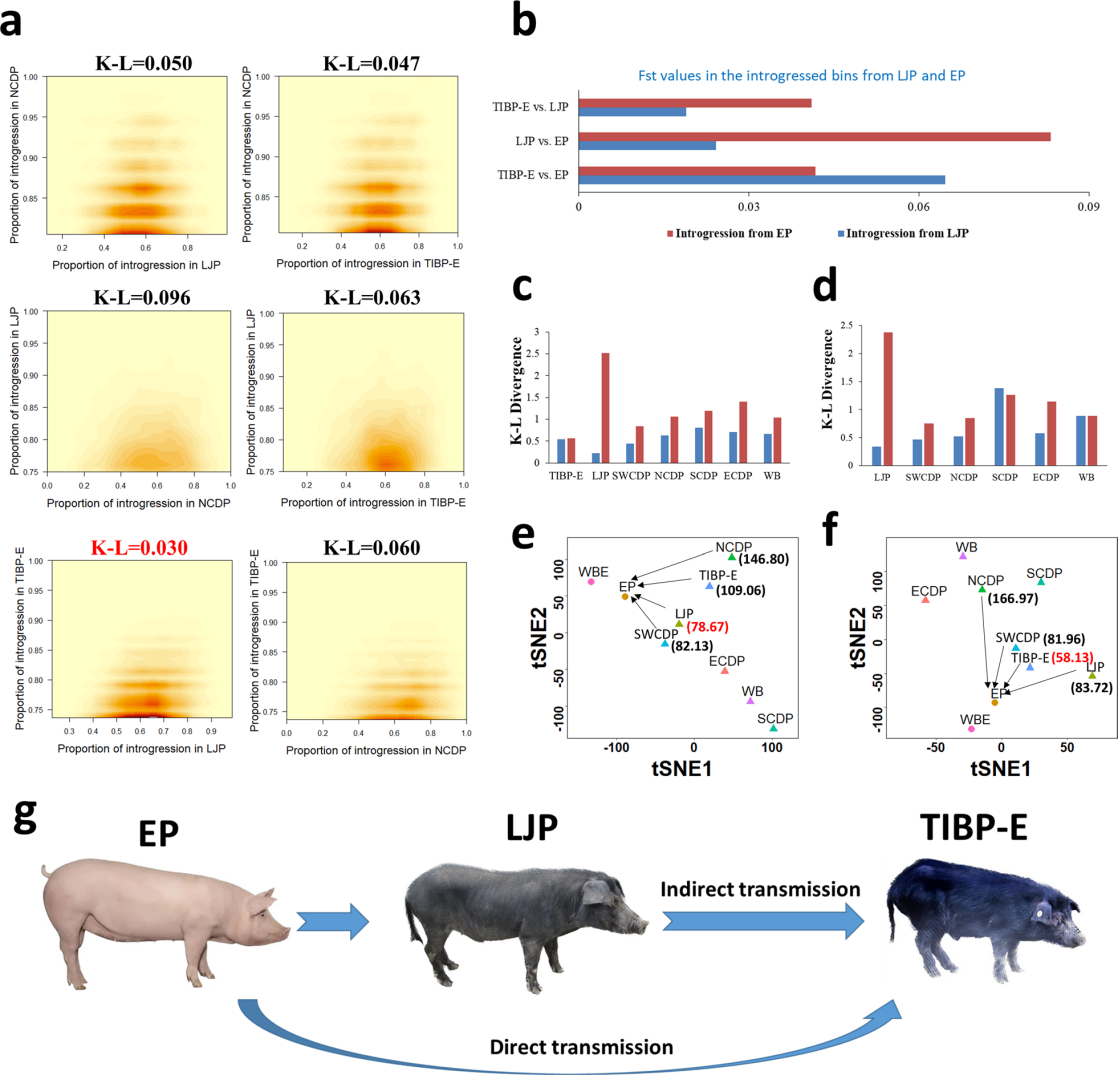
Genetic evidence of LJP as a potential mediator of EP haplotype transmission to Tibetan pigs. **a** Heatmap of kernel density for the proportion of introgression from EP into LJP, TIBP-E and NCDP based on the region of significant EP introgression bins in NCDP (upper panel), LJP (middle panel) and TIBP-E (lower panel); the K–L divergence is labelled at the top of each plot. **b** Average Fst values in the introgression bins from LJP and EP. **c** K–L divergence based on haplotype frequency in EP and Chinese indigenous populations in the introgression bins from EP into TIBP-E, including indirect (blue bars) and direct introgression (red bars). **d** K–L divergence based on haplotype frequency in TIBP-E and other Chinese indigenous populations in the introgression bins from EP into TIBP-E, including indirect (blue bars) and direct introgression (red bars). **e** and **f** t-distributed stochastic neighbour embedding (t-SNE) plot based on haplotype frequency in the bins of indirect and direct introgressed regions from EP; the numbers next to the populations indicate the Euclidean distance between EP and the respective indigenous Chinese populations (NCDP, SWCDP, TIBP-E, and LJP). **g** Schematic diagram of hypothetical introgression from EP to Tibetan pigs, including direct and indirect introgression and LJP as a potential mediator

Figure 2c shows that the number of overlapping EP-introgressed window bins was larger between LJP and TIBP-E (3186) than between LJP and NCDP (1826) or between TIBP-E and NCDP (2945). In addition, the distribution of EP-introgressed proportions in LJP was more similar to that in TIBP-E than in NCDP, indicating a more consistent variation of EP introgression proportions among the populations between LJP and TIBP-E than between LJP and NCDP, or between TIBP-E and NCDP. To validate the similarity between EP introgression for LJP and TIBP-E, we performed kernel density estimations and found that LJP and TIBP-E showed the most concentrated kernel density distribution in the significant EP-introgressed regions (Z-score > 2) of TIBP-E (0.23–0.99) (Fig. 3a), while the other groups showed more dispersed distributions (LJP vs. NCDP: 0.13–0.99; TIBP-E vs. NCDP: 0.08–1.00; NCDP vs. LJP: 0.00–1.00; TIBP-E vs. LJP: 0.08–1.00; and NCDP vs. TIBP-E: 0.00–1.00). Based on the EP-introgressed proportions, we found that the K–L divergence was lowest between TIBP-E and LJP (0.03, TIBP-E as the target group) (Fig. 3a). This indicates that the variation in EP introgression between LJP and TIBP-E was more similar in the EP-introgressed region of TIBP-E than in the other groups, which is consistent with the distribution of EP introgression proportions between LJP, NCDP, and TIBP-E (Fig. 2c). Then, we used LJP and SCDP as the reference population and TIBP-E as the target population and identified a large number of genomic segments introgressed from LJP, where 11,411 bins had an LJP introgression proportion greater than 95%, in contrast to the proportion of EP introgression in TIBP-E (57 bins), LJP (245 bins), and NCDP (550 bins). Among the 11,411 bins, 9909 were not identified as having a significant EP introgression in TIBP-E. Therefore, there may have been vast gene flows between LJP and TIBP-E, which may have driven transmission of EP introgressed segments from LJP into TIBP-E. We then used the Loter software to re-analyse the introgression bins from EP into LJP or TIBP-E. For the bins of introgression from EP into TIBP-E, we used LJP and EP as reference populations and TIBP-E as the target group and identified 51 bins as haplotypes from LJP other than EP (Z-score > 2). Conversely, for the introgression bins from EP into LJP, we obtained no significant TIBP-E introgression into LJP (see Additional file 1: Tables S6 and S7). Therefore, LJP-like haplotypes were identified as EP introgression in TIBP-E, but there were no significant TIBP-E haplotypes in LJP.

To further validate the potential EP introgression events that were mediated by LJP, we retrieved the top 51 bins identified as EP introgressions into TIBP-E (Z-score > 2) but not from LJP (Z-score < -2) for comparison (regarded as direct EP introgression), and also retrieved the top 51 bins identified as TIBP (Z-score > 2). The results showed discrepant genetic diversity among direct EP introgressions, indirect EP introgressions, and genomic segments of TIBP itself, with the introgression components of LJP (indirect EP introgression) displaying greater genetic diversity than those in TIBP, but a lower genetic diversity than the corresponding parts of EP (direct EP introgression) (Duncan test, p < 2e−16) (see Additional file 2: Fig. S8). To explore the genetic similarity of indirect EP introgression between LJP and TIBP-E, we computed the average Fst based on SNPs within the 51 introgression bins from LJP or EP that showed low differentiation between LJP and TIBP-E, LJP, and EP, but higher differentiation between TIBP-E and EP. This analysis showed that the average Fst value of TIBP-E vs. LJP was higher than that of introgression bins from LJP, higher in LJP vs. EP, and lowest in TIBP-E vs. EP (Fig. 3b). Based on haplotype frequencies in the 51 bins between EP and Chinese indigenous pigs, or between TIBP-E and other Chinese indigenous pigs, we found that the K–L divergence between EP and TIBP-E was similar when using the 51 introgression bins from EP or LJP, but different between EP and the other Chinese indigenous populations (Fig. 3c). When using introgression bins from LJP, we observed that LJP had the closest relationship with EP, even when compared with TIBP-E and EP. Based on introgression bins from EP, we observed that EP had a closer relationship with TIBP-E than with the other groups. We also compared the relationship between TIBP-E and other indigenous Chinese pig populations (Fig. 3d). For introgression bins from LJP, the K–L divergence was lower between LJP and TIBP-E than between the other groups, but was highest when using introgression bins from EP, and relatively high between TIBP-E and the others. T-SNE clustering analysis based on haplotype frequencies in the 51 bins showed that, for introgression bins from LJP, the Euclidean distance was lower between EP and LJP (78.8) than between other groups, reflecting their close relationship (Fig. 3e); for introgression bins from EP, TIBP-E was the most closely related group (Euclidean distance = 58.1) (Fig. 3f). Maximum-likelihood trees based on SNPs within the two groups of bins showed consistent results (see Additional file 2: Fig. S9).

### EP introgression in relation to thoracic vertebra number in LJP

**Fig. 4:**
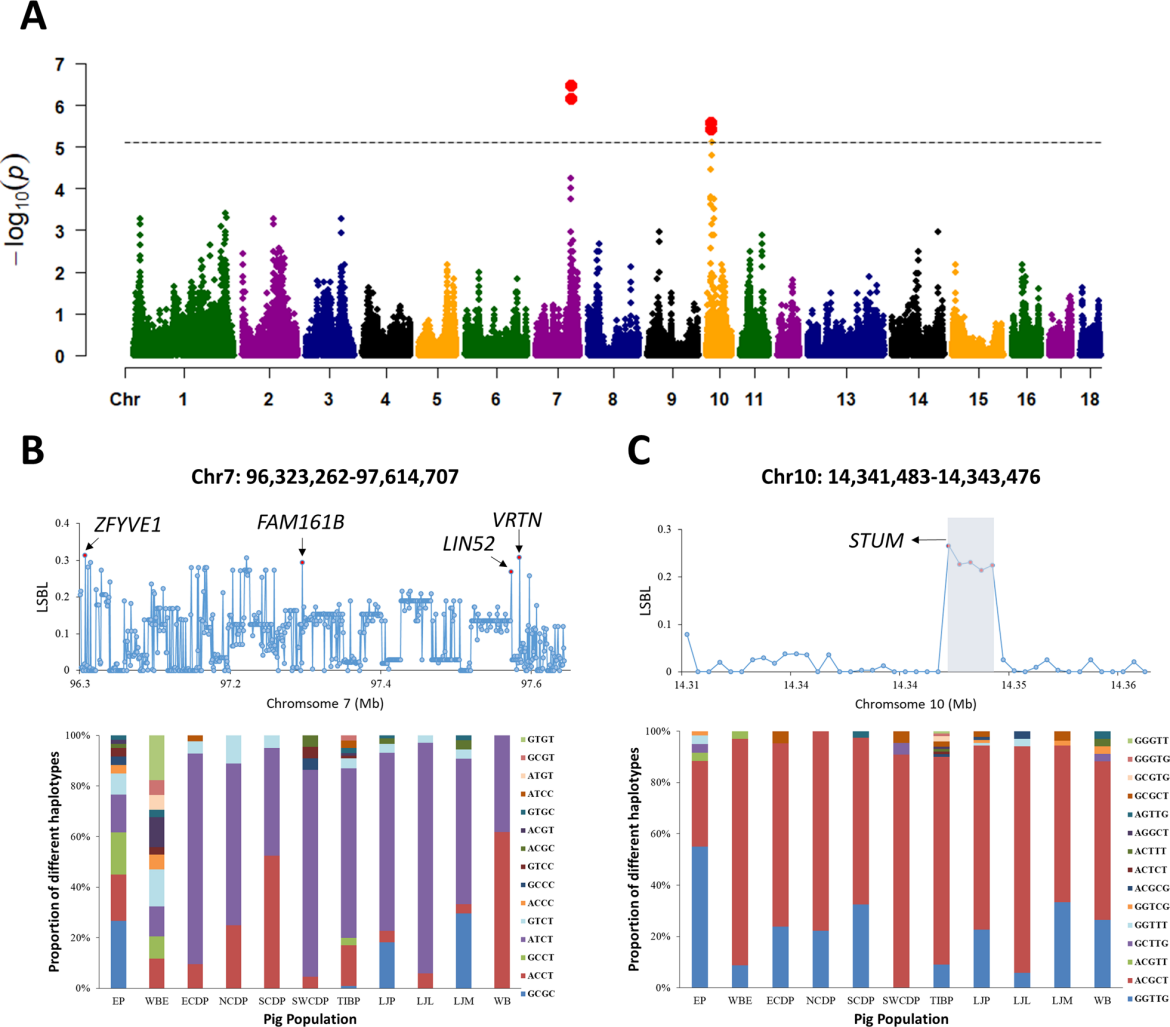
Genetic analysis of differences in degree of EP introgression between LJP with fewer (LJL) or more (LJM) thoracic vertebrae. **a** Manhattan plot of introgression differences related to thoracic vertebra number in LJP, where the horizontal grey dashed line indicates the whole-genome significance threshold and the red dots represent the significant window bins (Fisher-test, FDR < 0.05). **b** and **c** LSBL values (upper panel) in the significant introgressed regions from EP on chromosomes 7 and 10; the red dots indicate the SNP sites with the highest LSBL values in high LD and the respective genes are labelled above the plot. The respective haplotype frequencies (lower panel) on chromosomes 7 and 10 in each population

EP introgression may have affected the performance of indigenous Chinese pigs for economically important traits. In this study, we investigated whether EP introgression was related to the number of thoracic vertebrae in LJP by comparing a group from the LJP population with fewer thoracic vertebrae (LJL, < 14, n = 17) with another group with more thoracic vertebrae (LJM, > 14, n = 27) (see Additional file 1: Table S1 and Additional file 2: Fig. S10). Population admixture analyses revealed that the LJM group had a significantly higher EP composition (average: 19.3%) than the LJL group (average: 15.9%) when K = 6 (one-tailed t-test, p = 0.032). In addition, *D*-statistical analysis indicated that the LJM group shared a larger number of derived alleles with EP than the LJL group (D = 0.014, Z = 2.85). To further investigate this, we compared the EP-introgressed proportion between the LJM and LJL groups using 5-SNP window bins and identified 103 bins with a significant difference between these two populations (Fisher’s exact test, FDR < 0.05) (Fig. 4a and see Additional file 1: Table S8) on chromosomes 7 and 10. To further investigate which introgressed haplotype affects this trait, within these bins, we performed a locus-specific branch-length (LSBL) analysis for each SNP and found that the four SNPs with the highest LSBL values were in high LD (r^2^ > 0.8), spanning a 1.27 Mb region on chromosome 7 (Fig. 4b). Using these four sites as a haplotype block, we observed 11 haplotypes in the EP population. The dominant haplotype was ‘GCGC’, with a frequency of 26.7%, while indigenous Chinese pig populations shared a different dominant haplotype (ATCT: 3.7–52.5% or ACCT: 11.8–91.2%). The dominant EP haplotype (‘GCGC’) was present in the LJM group (frequency 29.6%), but rarely in the LJL group (dominant haplotype ATCT: 91.2%; EP-dominant haplotype: 0.0%) and other Chinese pig groups (EP dominant haplotype: 0.0–1.0%). Furthermore, individuals that carried the EP dominant haplotype had a significantly larger number of vertebrae than the other individuals (one-tailed t-test, p = 1e−4) (see Additional file 2: Fig. S11), indicating that EP introgression had a significant influence on thoracic vertebrae number in LJP. The related genes included *ZFYVE1*, *FAM161B*, *LIN52*, and *VRTN*. We also identified five SNPs (r^2^ > 0.7) with the highest LSBL values in the EP-introgressed region on chromosome 10 (Fig. 4c), spanning 1.99 kb in the 3′-UTR region of the *STUM* gene. Haplotype frequency analysis showed that the dominant haplotype in the Chinese pig population was ‘ACGCT’ (65.0–90.9%), while in EP it was 'GGTTG' (55.0%). The frequency of the dominant haplotype from EP was higher in the LJM group (33.3%) than in the LJL group (5.9%), and individuals carrying 'GGTTG' had a significantly larger thoracic vertebra number than the other individuals (one-tailed t-test, *p* = 0.001) (see Additional file 2: Fig. S11).

## Discussion

### Genetic structure of LJP and genetic introgression from European breeds

We investigated the genetic structure of LJP and its relationship with other pig populations. Our findings revealed that LJP formed a distinct branch in the phylogenetic tree, and has close genetic similarity to breeds that are present in the Southwest of China (SWCDP and TIBP) (Fig. 1 and see Additional file 2: Figs. S1–S3). However, the admixture analysis indicated that LJP had genetic components from European commercial lines, as well as from certain other Chinese domestic pigs (Fig. 1c). To further investigate the introgression signals from EP, we used outgroup*-f*3, *f*4, *D* statistics, and topology analyses. Our results confirmed introgression from EP into multiple indigenous Chinese breeds, suggesting that EP introgression events could have been more extensive than what we have discovered in this study.

In recent decades, the introduction of commercial European pig breeds to rural areas in China has contributed to economic growth, especially with the development of regional road infrastructure. This has led to an update in the genetic resources of Chinese domestic pigs. Previous studies have found genetic components from EP in Chinese breeds, [11, 14–16, 40, 41], but EP introgression into LJP (a specific Chinese pig populations) has not been studied. Our study confirms the presence of EP introgression into multiple Chinese pig populations, including NCDP, SWCDP, TIBP-E, and LJP. However, there are differences in the extent of EP introgression among these populations (Fig. 2c–f). Specifically, we observed that LJP has the shortest introgressed blocks compared to TIBP-E and NCDP (Fig. 2a and b). This suggests that the EP introgressed segments have not undergone strong selection, possibly because these pigs were primarily raised in free-ranging herds alongside other local livestock and were not subjected to intensive breeding. For NCDP, we observed a higher density of EP introgression bins, with a high proportion of introgressed segments (a thicker density at the right tail of the density plot of EP introgressed proportion) in this population than in LJP and TIBP-E (Fig. 2c–f). In addition, we identified 'fixed' bins (i.e. with all individuals sharing the same EP haplotype) among significant introgression bins from EP into NCDP (Fig. 2e), and even for significant introgression bins from EP into TIBP-E, we observed 'fixed' bins for NCDP (Fig. 2f), which suggests that EP introgression into NCDP was strongly selected for, while EP introgressed bins in LJP and TIBP-E were not as strongly selected for. Although we also observed larger introgressed blocks for TIBP-E and NCDP compared to LJP, the results of the NJ tree and population structure analysis (Fig. 1b, and see Additional file 2: Fig. S3) suggested that the large EP-introgressed blocks in TIBP-E were introduced by recent hybridisation between TIBP-E and EP, rather than being due to strong selection. This is consistent with previous documentation of interbreeding between Tibetan and European pigs or other SWCDP at the end of the last century [12]. In conclusion, LJP has a close genetic relationship with pig breeds distributed in the Southwest of China and the EP introgression into this population differs from that into other indigenous Chinese breeds and may not have been strongly selected for.

### Comparative analysis of EP introgression into LJP and other populations suggests multi-step introgression of commercial European lines into Chinese indigenous pigs

The history of hybridization in indigenous Chinese pig breeds is complex, with dominant haplotypes from a given population potentially transmitted through multiple steps into another population. A two-step introgression from large-sized pigs into small-sized pigs to produce the Dahuabai breed is consistent with historical accounts [6]. The gene flow event between EP and indigenous Chinese pigs is likely more recent and widespread than the introgression into the Dahuabai breed, mainly due to improved road access to the outside world compared to that in Ancient China. In the 1970s and 1980s, China began importing multiple commercial European pig lines to improve meat production of indigenous Chinese pigs, leading to the widespread interbreeding between European and Chinese breeds. In certain regions, interbreeding occurred among Chinese pig breeds, alongside hybridization with EP, which facilitated the transmission of European haplotypes to other Chinese pig populations.

In our study, we initially observed a higher degree of similarity in the distribution of EP introgression proportion in LJP and TIBP-E than in NCDP (Fig. 2c). This led us to consider two possibilities: (1) the same or similar Western pigs were introgressed into both pig breeds, or (2) the transmission of EP introgressed segments between LJP and TIBP-E. We further identified a large number of genomic segments that were introgressed from LJP, most of which were not detected as EP introgression into TIBP-E. Of the 11,411 bins, 9909 were not identified as having a significant EP introgression proportion in TIBP-E. This suggests extensive gene flow between LJP and TIBP-E, which may have facilitated the transmission of EP introgressed segments from LJP into TIBP-E. The *D*-statistics and Loter analyses (see Additional file 1: Tables S6 and S7) confirmed the presence of LJP-like haplotypes as well as EP introgression into TIBP-E, while no significant TIBP-E haplotypes were found in LJP. Based on these findings, we propose two scenarios: (1) EP haplotypes were directly transmitted to TIBP-E, or (2) LJP or other Chinese indigenous breeds served as intermediaries for the transmission of EP haplotypes to TIBP-E, involving both one-step and multiple-step EP introgressions. To test this hypothesis, we further compared direct and indirect EP introgression (from LJP), and also compared genomic segments of TIBP with no significant introgressed signals from EP and LJP. According to the comparison analysis of average site-pi of SNPs located within the bins of direct EP introgression, indirect EP introgression, and of genomic segments from TIBP in TIBP-E, we observed that the genomic segments of indirect EP introgression displayed a higher genetic diversity compared to those from TIBP, but lower than the genomic segments from EP (direct EP introgression) (see Additional file 2: Fig. S8). These results suggest that LJP or other pigs in the Southwest of China may have served as pig breeds for transmitting introgressed EP segments to Tibetan pigs. To explore the genetic similarity of genomic segments in the region of indirect EP introgression between TIBP-E and LJP, we compared Fst values and K-L divergence between TIBP-E and other pig populations (Fig. 3a–d), and observed the lowest level of genetic differentiation and K-L divergence between TIBP-E and LJP. The results of T-SNE and Treemix further confirmed a close relationship between LJP and TIBP-E when based on bins of EP introgression into TIBP-E (Fig. 3e and f and see Additional file 2: Fig. S9), suggesting that LJP played a potential role in transmitting EP haplotypes to Tibetan pigs (Fig. 3g).

Our finding that the transmission of EP haplotypes to TIBP-E was likely mediated by LJP, with no significant transmission observed from TIBP-E to LJP implies that the introgression of EP was one-way, from LJP into TIBP-E, rather than the other way around. We hypothesize that male LJP pigs were introduced into the TIBP-E population at some point, leading to indirect EP introgression. However, it is also possible that this transmission occurred from other LJP-like pigs in Southwestern China, as we observed a relatively close relationship between SWCDP and EP based on the EP introgressed region of TIBP-E (Fig. 3f and see Additional file 2: Fig. S9). Our findings are also consistent with the historical fact that TIBP began mixing with other breeds at the end of the last century, specifically with the TIBP population located near the riverdale or highway that connected them to the outside world in Southwestern China [12]. Furthermore, multiple breeds, including LJP, could collectively have contributed to the transmission of European haplotypes to TIBP. The complexity of recent transmissions has made it challenging to trace exact ancestry, but we were able to roughly distinguish ancestry based on introgression level and genetic similarity between TIBP-E and other Chinese pig population, and identify the potential breeds for transmitting EP introgressed haplotypes. To further investigate the transmission network of European haplotypes in indigenous Chinese breeds, future studies should include a larger number of breeds and populations and conduct coalescent simulation for building a suitable demographic model to confirm the breeds that transmit genomic segments from EP and to infer the transmission network precisely.

### EP introgression in relation to phenotypic changes in LJP

Investigation of the association between EP introgression of segments on chromosomes 7 and 10 and the number of thoracic vertebrae in LJP identified variants in the *VRTN* and *STUM* genes that are associated with this trait. *VRTN* is a well-known gene involved in somite formation in animals [42], while previous research has shown that the *STUM* gene is required for the transduction of mechanical stimuli in proprioceptive neurons that sense joint angles in Drosophila [43]. In addition, network analysis using the STRING human database revealed that *STUM* is co-expressed with *CPNE7* and *LRRC5*, which are involved in hard tissue regeneration and stress fracture predisposition, respectively (see Additional file 2: Fig. S12) [44, 45]. These findings suggest that EP introgression may have had a positive effect on the performance of economic traits. However, it should be noted that the observed introgression pattern is a result of both drift and selection and the actual impact on a trait depends on the intensity of selection on that trait. We were unable to evaluate the effect of selection for thoracic vertebra number in the 44 LJP pigs evaluated here because they were intentionally selected from a population with extreme differences for thoracic vertebrae number. However, the shorter length (than for NCDP and TIBP-E) of the introgressed segments suggests that EP introgressed segments may not have been strongly selected for in the LJP population. It is worth noting, however, that EP introgression is not limited to the LJP breed, as numerous breeds in China have undergone EP introgression to improve productivity, which may have led to increased phenotypic variation in other indigenous Chinese breeds. Further analysis is needed to explore the association between phenotypic changes and EP introgression in other domestic Chinese pigs.

## Conclusions

The results of this study lead to several conclusions: (1) we show that LJP shares a close genetic relationship with pigs distributed in Southwest China; (2) we report genetic evidence of introgression from EP into the LJP breed, with varying levels of EP introgression observed among LJP and other Chinese breeds, and we suggest that the LJP pigs were a potential mediator for transmitting EP haplotypes; and (3) we show that there is a correlation between introgression from EP and the number of thoracic vertebrae in LJP. Collectively, our findings provide genetic evidence for recent introgression from commercial European lines into the LJP breed, which likely contributes to phenotypic changes in economic traits.

### Supplementary Information

**Additional file 1: Table S1.** Samples used in this study. The data include sample information, quality control data, phenotypic data. **Table S2.** *D*-statistic and *f*4-ratio results. The table presents *D*-statistic, Z-transformed *D*-statistic, p-value (for *D*-statistic),* f*4-ratio, ABBA and BABA values for all pig groups. **Table S3.** Significant EP introgression window bins in NCDP. The table presents the detected EP introgression bins and proportions in NCDP. **Table S4.** Significant EP introgression window bins in TIBP-E. The table presents the detected EP introgression bins and proportions in TIBP-E. **Table S5.** Significant EP introgression window bins in LJP. The table presents the detected EP introgression bins and proportions in LJP. **Table S6.** Potential indirect introgression from EP into TIBP-E. The table presents the indirect EP introgression window bins in TIBP-E, which were detected as introgression from LJP. **Table S7.** Potential direct introgression from EP into TIBP-E. The table presents the direct EP introgression window bins in TIBP-E. **Table S8.** Fisher-test of EP introgression proportion in LJL and LJM. The table presents the Fisher-test of EP introgression proportion in LJL and LJM, and the significant level that was defined as corrected p-value FDR < 0.05.

**Additional file 2: Figure S1.** Principal component analysis (PCA) of pig breeds using whole-genome SNPs. PC1, PC2, and PC3 explained 6.24%, 2.58%, and 1.60% of the genetic variance, respectively. **Figure S2.** Heatmap based on the IBS distance matrix of all pigs. The figure presents the IBS distance matrix of all pigs without an outgroup (n = 228). **Figure S3.** Neighbour-joining (NJ) tree and heatmap based on the p-distance matrix of all pigs. NJ tree and pairwise relationship-based the p-distance matrix (n = 228). **Figure S4.** Cross-validation (CV) error of the Admixture results for K = 2 to 10. The red dot represents the lowest CV error (K = 6, CV error = 0.512). **Figure S5.** Gene flow analysis between EP and indigenous Chinese pigs. (**a**) *D*-statistics with D (sister group, target group, EP, Outgroup) (y-axis) to detect introgression into the target group from EP, where the Outgroup was Sumatran pig, the x-axis represents the sister group with no significant EP introgression (GZTIB, LTTIB, and SLTIB), and the lines in the legend with different colours depict the respective target groups. (**b**) Outgroup-*f*3 statistics for all indigenous Chinese populations and EP, i.e. *f*3 (Chinese indigenous population, EP; Outgroup). **Figure S6.** Mean weightings for the three possible taxon topologies across the whole genome, the topologies for the four taxa as rooted, with Sumatran wild boars as the outgroup. (**a**) Topology analysis for LJP, SCDP, and EP. (**b**) Topology analysis for NCDP, SCDP, and EP. (**c**) Topology analysis for TIBP-E, SCDP, and EP. (**d**) Topology analysis for SWCDP, SCDP, and EP. **Figure S7.** Total size of introgression blocks from European pigs (EP) into Northern Chinese domestic pigs (NCDP), Lijiang pigs (LJP), and Tibetan (specifically Diqing and Daocheng) pigs (TIBP-E) after down-sampling for LJP (n = 18). To exclude the effect of sample size, LJP was down-sampled to 18. **Figure S8.** Site-pi of SNPs in the region of 51 window bins for direct introgression (DI), indirect introgression (IDI) and for genomic segments from TIBP in TIBP-E. a, b, c represent the significance for Duncan’s test of DI, IDI, and TIBP (p < 2.2e-6), respectively. **Figure S9.** Maximum-likelihood tree of single nucleotide polymorphisms (SNPs) in the bins of indirect (a) and direct (b) introgressed regions from EP. The maximum-likelihood trees based on SNPs located within the bins identified as introgression from EP or LJP. **Figure S10.** Summary of thoracic vertebra numbers. LJP were grouped according to the number of thoracic vertebrae, including a group of individuals with fewer thoracic vertebrae (LJL, less than 14, n = 17) and a group of individuals with more thoracic vertebrae (LJM, more than 14, n = 27). **Figure S11.** Boxplot of thoracic vertebrae number based on haplotypes on chromosomes 7 (region: Chr7-96323262_97614707) and 10 (region: Chr10-14341483_14343476). The region represents the haplotype block with a high LD level, including blocks on chromosomes 7 and 10. **Figure S12.** Interaction network centred by the STUM protein. The interaction network of STUM analysis was based on the STRING human database, where CPNE7 and LRRC5 were identified as co-expressed proteins.

## Data Availability

Raw sequencing data that support the findings of this study have been deposited in the NCBI database under accession number PRJNA942216. The benefits of this research accrue from the sharing of our data and results in public databases as described above.
