# Supplementary material for: Genetic introgression from commercial European pigs to the indigenous Chinese Lijiang breed and associated changes in phenotypes
Source: Genet Sel Evol. 2024 Apr 2;56:24. doi: 10.1186/s12711-024-00893-8 (PMC10985947; doi:10.1186/s12711-024-00893-8)
Supplement: Supplementary file 2 — Additional file 2: Figure S1. Principal component analysis (PCA) of pig breeds using whole-genome SNPs. PC1, PC2, and PC3 explained 6.24%, 2.58%, and 1.60% of the genetic variance, respectively. Figure S2. Heatmap based on the IBS distance matrix of all pigs. The figure presents the IBS distance matrix of all pigs without an outgroup (n = 228). Figure S3. Neighbour-joining (NJ) tree and heatmap based on the p-distance matrix of all pigs. NJ tree and pairwise relationship-based the p-distance matrix (n = 228). Figure S4. Cross-validation (CV) error of the Admixture results for K = 2 to 10. The red dot represents the lowest CV error (K = 6, CV error = 0.512). Figure S5. Gene flow analysis between EP and indigenous Chinese pigs. (a) D-statistics with D (sister group, target group, EP, Outgroup) (y-axis) to detect introgression into the target group from EP, where the Outgroup was Sumatran pig, the x-axis represents the sister group with no significant EP introgression (GZTIB, LTTIB, and SLTIB), and the lines in the legend with different colours depict the respective target groups. (b) Outgroup-f3 statistics for all indigenous Chinese populations and EP, i.e. f3 (Chinese indigenous population, EP; Outgroup). Figure S6. Mean weightings for the three possible taxon topologies across the whole genome, the topologies for the four taxa as rooted, with Sumatran wild boars as the outgroup. (a) Topology analysis for LJP, SCDP, and EP. (b) Topology analysis for NCDP, SCDP, and EP. (c) Topology analysis for TIBP-E, SCDP, and EP. (d) Topology analysis for SWCDP, SCDP, and EP. Figure S7. Total size of introgression blocks from European pigs (EP) into Northern Chinese domestic pigs (NCDP), Lijiang pigs (LJP), and Tibetan (specifically Diqing and Daocheng) pigs (TIBP-E) after down-sampling for LJP (n = 18). To exclude the effect of sample size, LJP was down-sampled to 18. Figure S8. Site-pi of SNPs in the region of 51 window bins for direct introgression (DI), indirect introgression [file 12711_2024_893_MOESM2_ESM.docx]

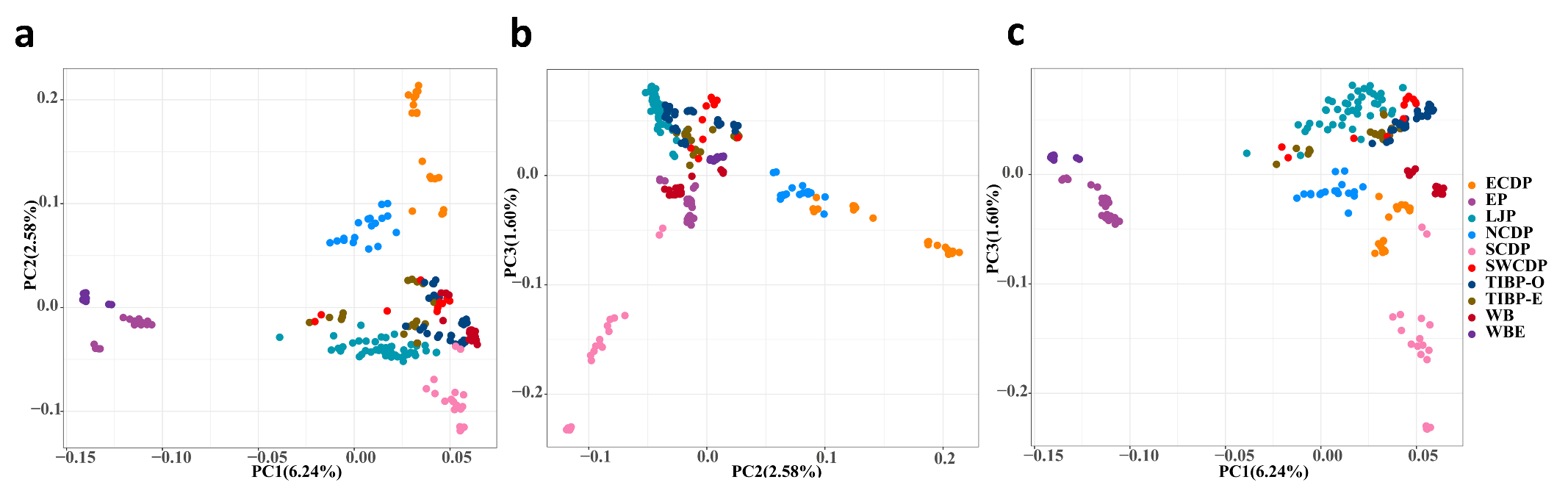


**Figure S1** Principal component analysis (PCA) of pig breeds using whole-genome SNPs.


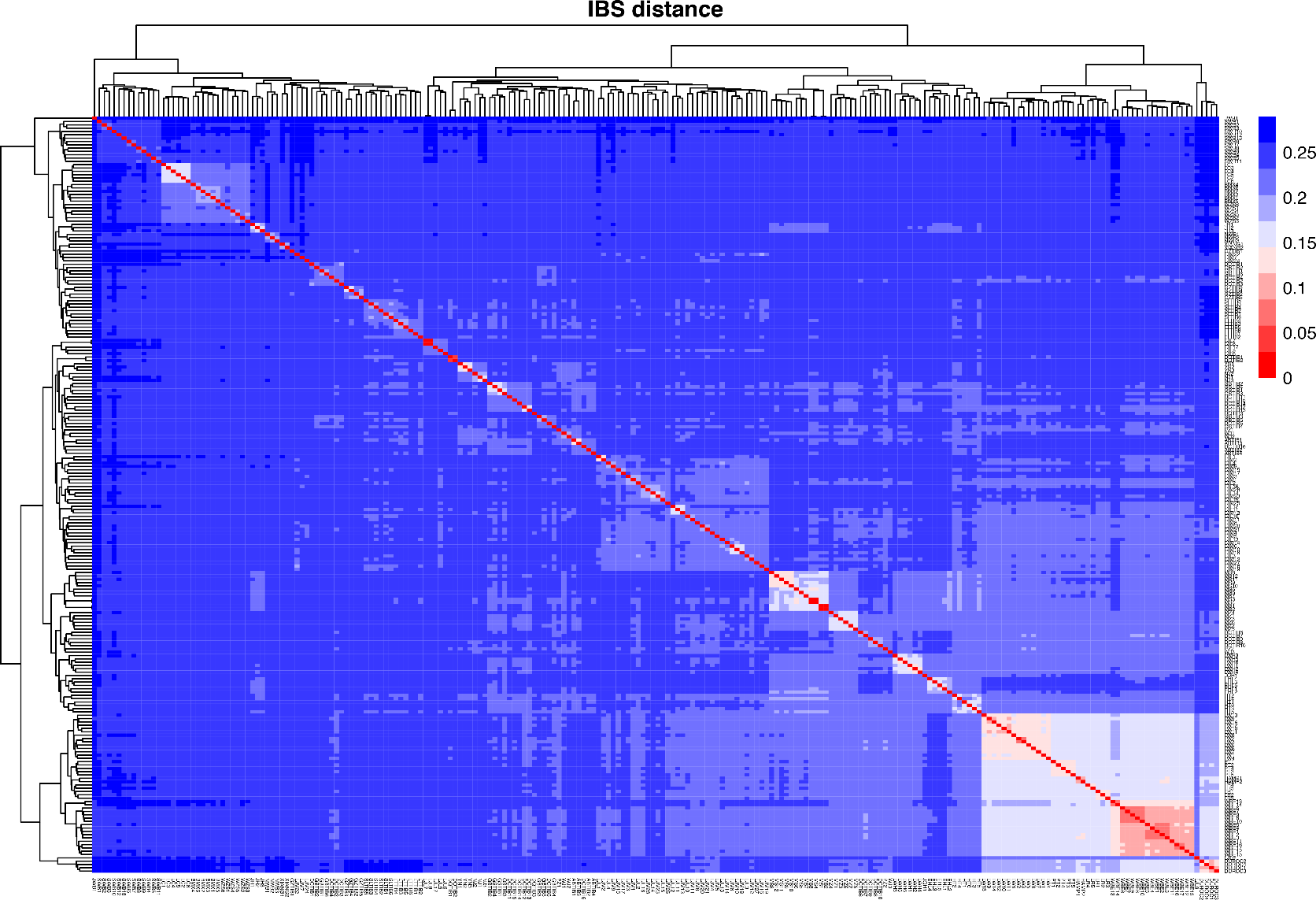


**Figure S2.** Heatmap based on the IBS distance matrix of all pigs.


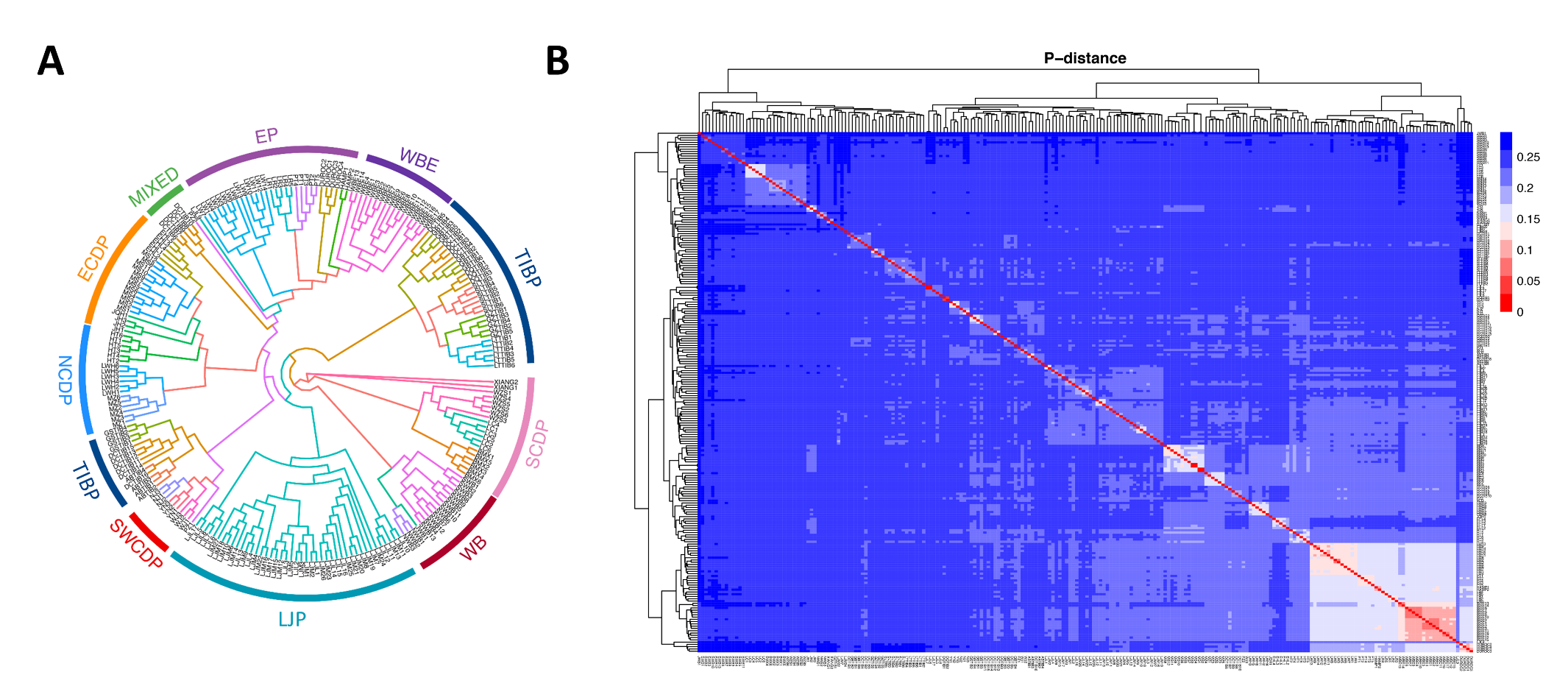


**Figure S3.** Neighbour-joining (NJ) tree and heatmap based on the p-distance matrix of all pigs.


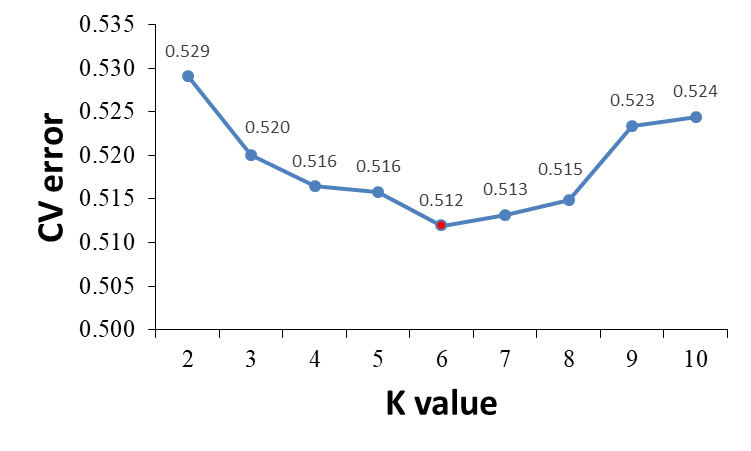


**Figure S4.** Cross-validation (CV) error of Admixture results for K = 2 to 10.


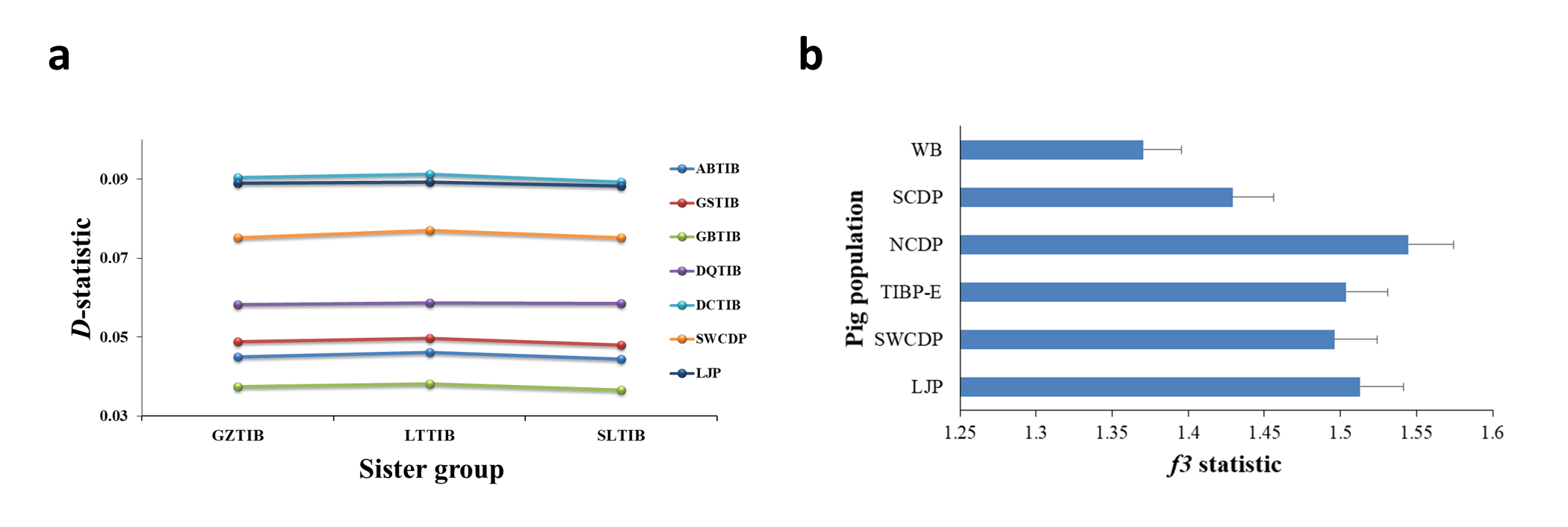


**Figure S5.** Gene flow analysis between EP and indigenous Chinese pigs.


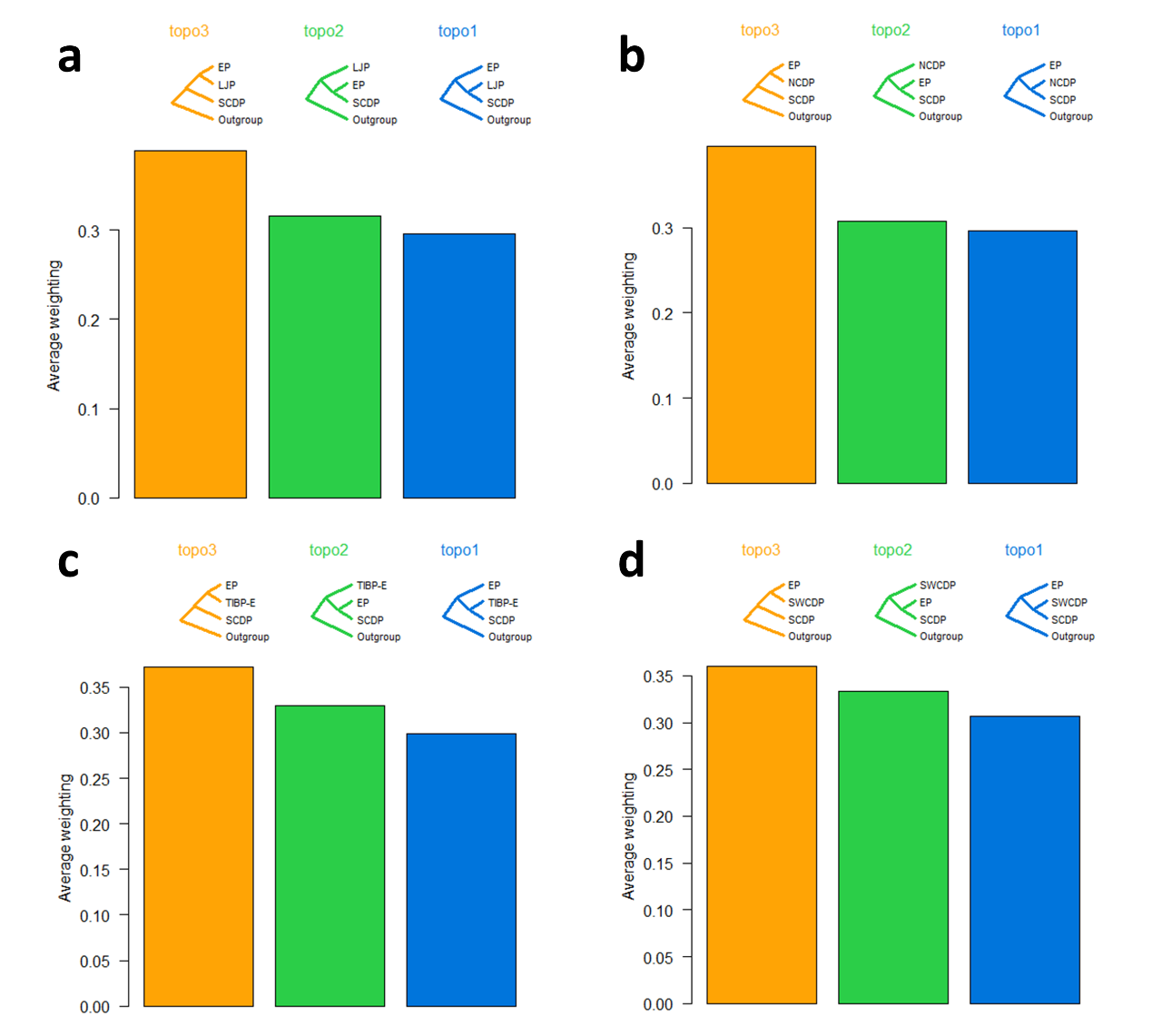


**Figure S6.** Mean weightings for the three possible taxon topologies across the whole genome, the topologies for the four taxa as rooted, with Sumatran wild boars as the outgroup.


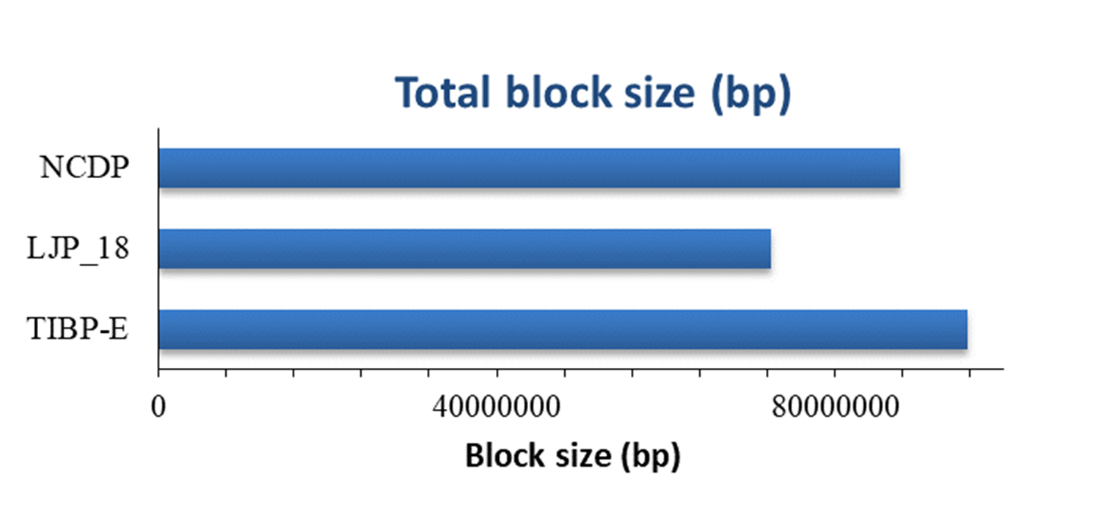


**Figure S7.** Total block size of introgression from European pigs (EP) to Northern Chinese domestic pigs (NCDP), Lijiang pigs (LJP), and Tibetan (specifically Diqing and Daocheng) pigs (TIBP-E) after down-sampling for LJP (n = 18).


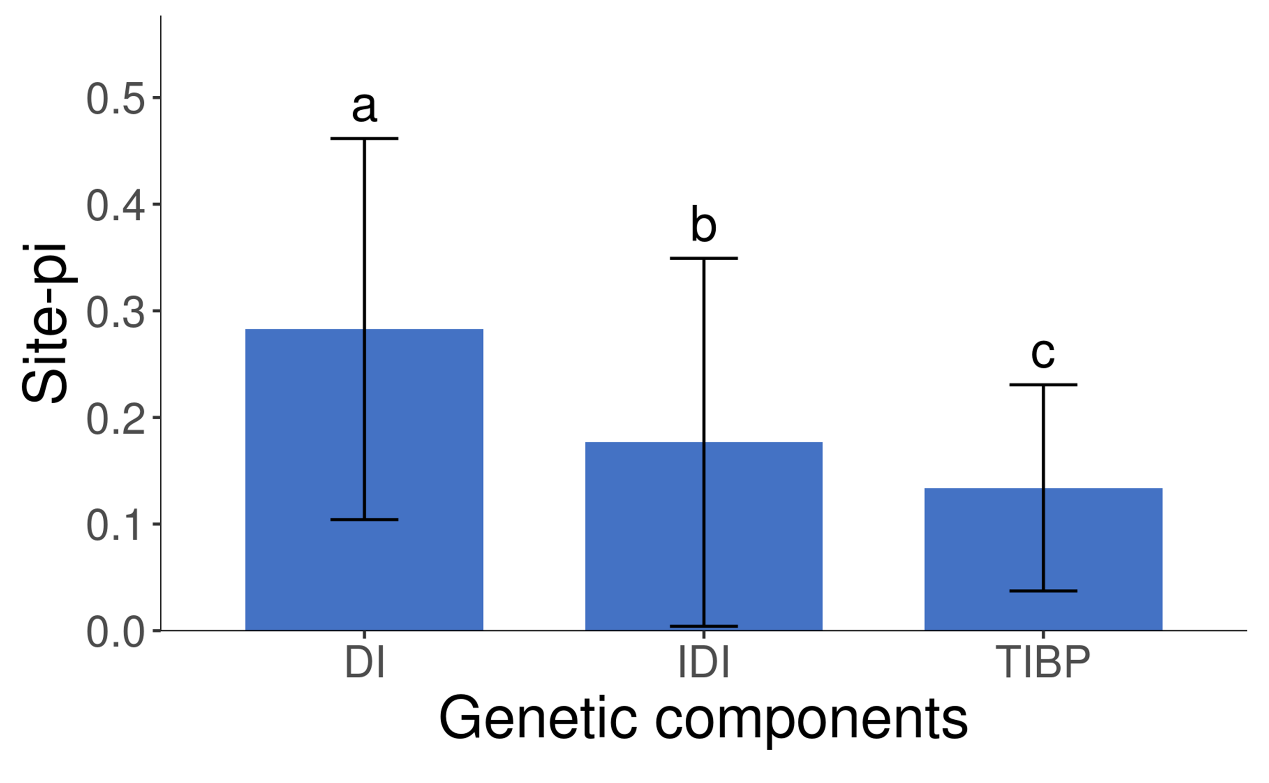


**Figure S8.** Site-pi of SNPs in the region of 51 window bins for direct introgression (DI), indirect introgression (IDI) and genomic segments of TIBP itself.


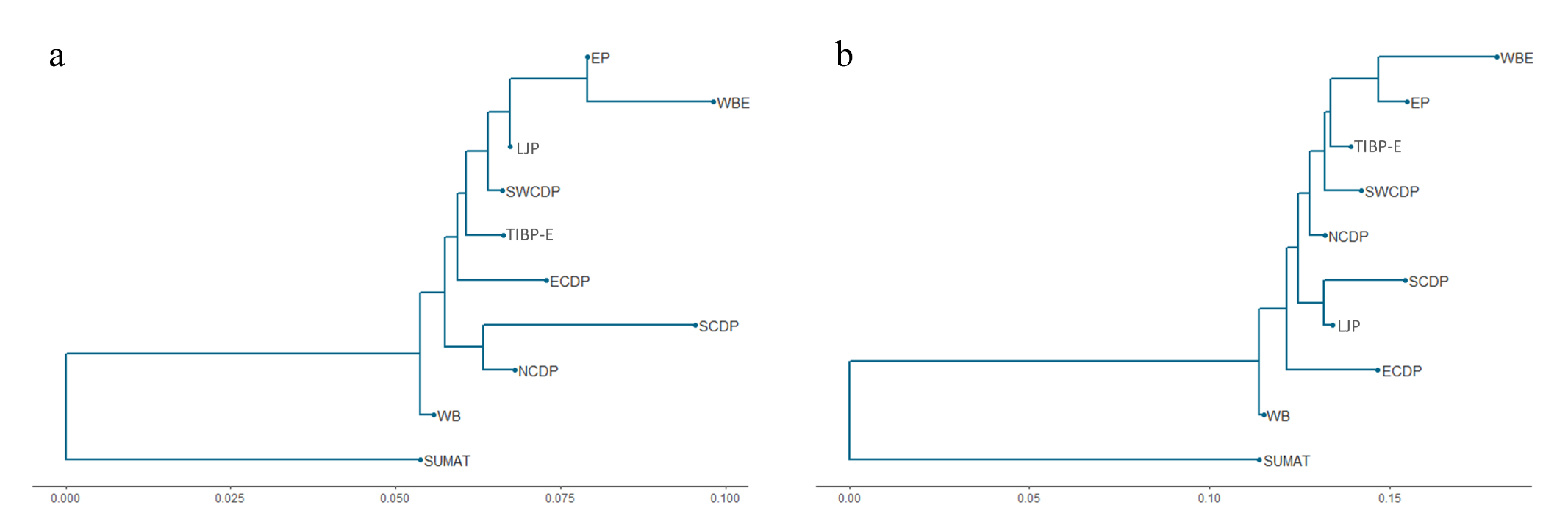


**Figure S9.** Maximum-likelihood tree of single nucleotide polymorphisms (SNPs) in the bins of indirect (a) and direct (b) introgressed regions from EP.


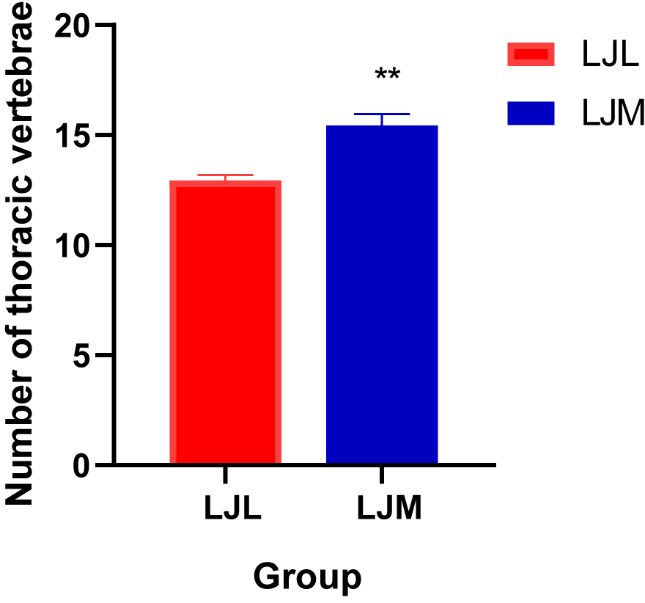


**Figure S10.** Summary of thoracic vertebra numbers.


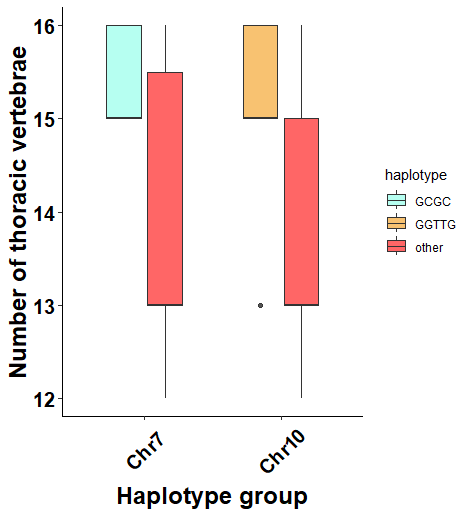


**Figure S11.** Boxplot of thoracic vertebrae number based on haplotypes on chromosomes 7 (region: Chr7-96323262_97614707) and 10 (region: Chr10-14341483_14343476).


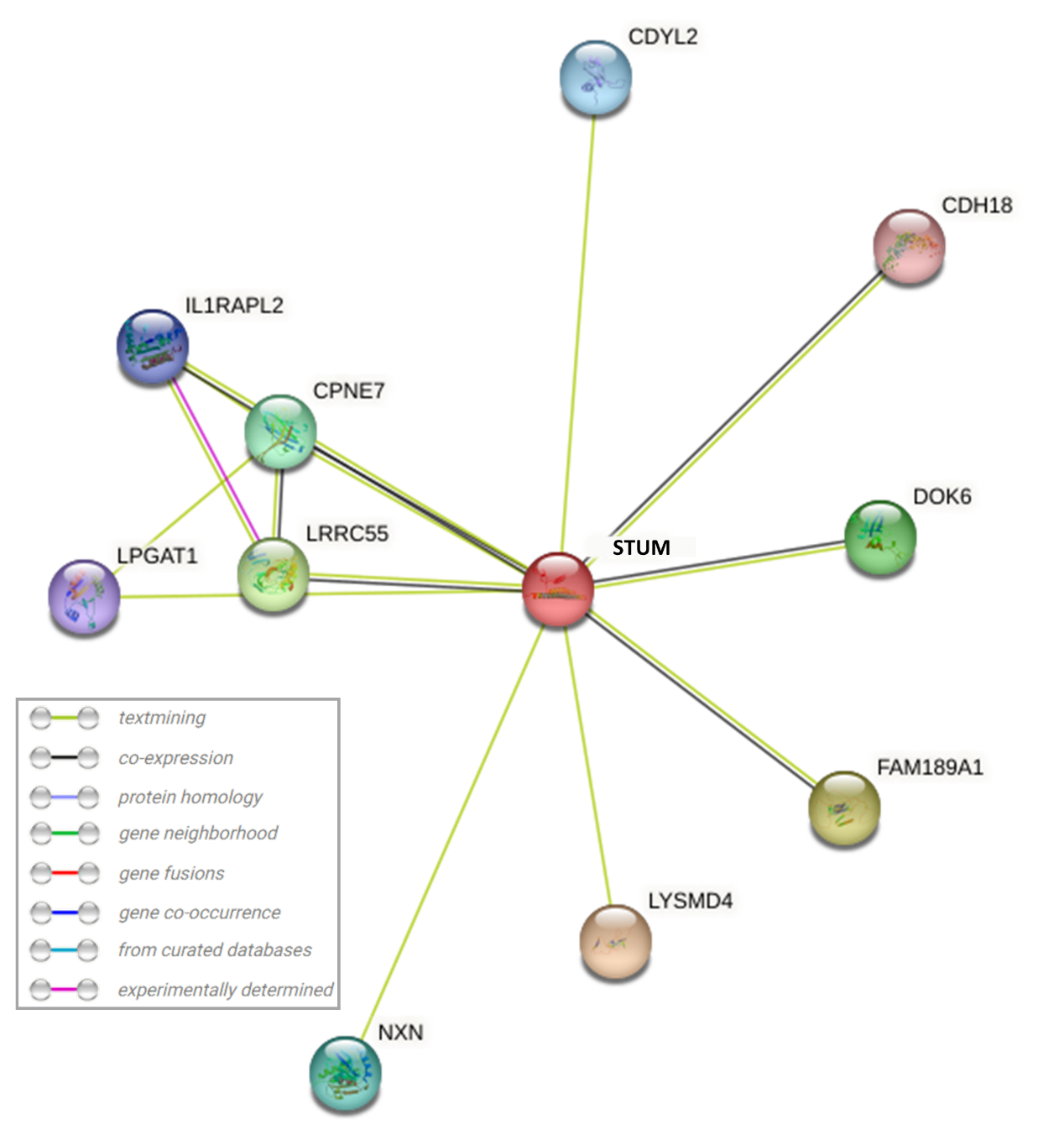


**Figure S12. I**nteraction network centred by STUM.
